# Evaluation of posterior capsule opacification of the Alcon Clareon IOL vs the Alcon Acrysof IOL using a human capsular bag model

**DOI:** 10.1186/s12886-020-01349-5

**Published:** 2020-02-27

**Authors:** Anna Hillenmayer, Christian M. Wertheimer, Stefan Kassumeh, Annabel von Studnitz, Nikolaus Luft, Andreas Ohlmann, Siegfried Priglinger, Wolfgang J. Mayer

**Affiliations:** Cell and molecular biology research laboratory, Division of Ophthalmology, Ludwig-Maximilians-University Munich, Mathildenstrasse 8, 80336 Munich, Germany

**Keywords:** Posterior capsule opacification, Cataract surgery, Capsular bag model, Clareon, AcrySof

## Abstract

**Background:**

Posterior capsule opacification (PCO) after cataract surgery is influenced by intraocular lens (IOL) design and material. The following is an ex vivo comparison of PCO between the Clareon vs. the AcrySof IOL in human capsular bags.

**Methods:**

Twenty cadaver capsular bags from 10 human donors were used, with the novel hydrophobic IOL (Clareon, CNA0T0) being implanted in one eye and the other eye of the same donor receiving the AcrySof IOL (SN60WF) following phacoemulsification cataract surgery. Five capsular bags of 3 donors served as controls without IOL. Cellular growth of lens epithelial cells was photo-documented daily. The primary endpoint was the time until full coverage of the posterior capsule by cells. Furthermore, immunofluorescence staining of capsular bags for the fibrotic markers f-actin, fibronectin, alpha smooth muscle actin, and collagen type 1 were performed.

**Results:**

The new Clareon IOL did not show any disadvantages in terms of days until full cell coverage of the posterior capsule in comparison to the AcrySof (*p* > 0.99). Both, the Clareon (*p* = 0.01, 14.8 days) and the AcrySof IOL (*p* = 0.005, 15.7 days) showed a slower PCO development in comparison to the control (8.6 days). The fibrotic markers f-actin, fibronectin, alpha smooth muscle actin, and collagen type 1 were equally distributed between the two IOLs and differed from the control.

**Conclusions:**

A comparable performance has been found in the ex vivo formation of PCO between the two IOLs. Long-term clinical studies are necessary to reach final conclusions.

## Background

Posterior capsule opacification (PCO) develops regularly after cataract surgery [1]. This unwanted wound healing reaction is thought to originate from lens epithelial cells that remain in the capsular bag after cataract surgery, especially on the inside of the anterior capsule and the capsular equator. After getting activated by several surgically and implant induced cytokines and growth-factors, cells undergo epithelial–mesenchymal transition to become myofibroblasts [2]. Those myofibroblasts proliferate and migrate on the posterior capsule towards the optical axis below the intraocular lens (IOL). Strong differentiation causes Elschnig pearls and Soemmering ring formations [3]. The condition results in increased light scattering and aberrations. Clinically, the patient is disturbed by reduced vision and increased straylight.

Recently the Clareon IOL (Alcon, Fort Worth, Texas, USA) was introduced. Its’ design is similar to the AcrySof IQ (Alcon, Fort Worth, Texas, USA), but the new material is made of a proprietary cross-linked acrylic optic biomaterial developed by combining a hydrophilic polymer (2-hydroxyethyl-methacrylate) and a hydrophobic component (phenylethyl acrylate) with a chemically bonded ultraviolet blocker, a blue-light filtering chromophore and a water content of 1.5%, which is supposed to show reduced glistening and surface inhomogeneities [4]. The producing company also claims a new precision edge design to be possible with this material, featuring a modified posterior square optic edge to minimize positive dysphotopsias and prevent PCO [5]. The AcrySof single-piece IOL has been introduced about 20 years ago, showing PCO with a consecutive Nd:YAG laser capsulotomy rate of slightly above 10% after 10 years of implantation [5]. The IOL has a sharp optic edge, which is interrupted at the haptic optic junction and is made of a hydrophobic acrylic polymer mix (phenylethyl methacrylate, phenylethyl acrylate, butanediol diacrylate) [6].

Both, the IOL material [7] and the edge design, play a critical role in PCO formation and rate [8]. The IOL materials’ influence is supposedly due to the different binding properties of extracellular matrix proteins like fibronectin and vitronectin, which promote cell growth [9]. With respect to PCO-preventive IOL design efforts, the sharp edge is intended to act as a mechanical barrier for lens epithelial cells. In this instance, the capsule is shrinking through peripheral fibrosis and wrapped tightly across the sharp edge [10]. The AcrySof IQ and the Clareon are different in IOL material and square edge design, with potential improvements towards the newly developed Clareon IOL.

The aim of this study was to compare the timing of ex-vivo PCO formation after implantation of the Clareon in comparison with the AcrySof IOL. PCO develops over several years, with incidence numbers increasing up to 9 years after implantation of the IOL [11]. As the Clareon is a newly introduced IOL, there is little data on long-term incidence. To obtain an early assessment of PCO formation, the human capsular bag model is commonly used [12, 13] to accelerate PCO formation ex vivo. Minor differences in PCO formation after implanting an IOL can be observed to test new IOL designs and types [14–16]. Posterior capsule opacification as a type of fibrosis cannot only be reduced to cellular growth. Several molecular changes in the lens epithelial cells are observed [2]. Therefore and to obtain a valid comparison between the two IOL designs different molecular markers for fibrosis have been stained.

## Methods

### Human capsular bag preparation

Human capsular bags were prepared with some modifications as described by Cleary G. et al. [17] In brief, 25 cadaver eyes from 13 human donors, not available for transplantation purposes (at the age of 61 ± 15 years), were obtained from the institute of forensic medicine (Ludwig-Maximilians-University Munich, Germany) and processed within 4 to 24 h of death. None of the donors had a known history of eye disease. All experimental procedures and the tissue harvesting process included proper consent and approval, complied with the Declaration of Helsinki and were approved by the institutional review board (LMU Munich: approval id 73,416). After standard phacoemulsification cataract surgery, a paired eye approach was followed. One eye was implanted with the AcrySof SN60WF IOL (Alcon, Fort Worth, TX, USA), whereas the other eye of the same donor received the Clareon, CNA0T0 (Alcon, Fort Worth, TX, USA). This was repeated for 10 consecutive donors. 5 eyes of 3 donors served as controls without implantation of an IOL. After implantation, an anterior corneoscleral ring was removed using a manual trephine (15 mm). In order to preserve normal capsular and zonular architecture, the IOL-capsule-zonule-ciliary body complex was dissected from the globe in one piece and, with the posterior capsule facing up, pinned to a soft silicone ring by entomological pins (Ento Sphinx, Pardubice, Czech Republic) through the ciliary body. The capsule was supported by the native zonules and suspended freely within culture medium.

### Cell culture and quantification of PCO

Specimens were cultured in cell culture plates (NUNC, Langenselbold, Germany). The culture medium (MEM Earles, Biochrom AG, Berlin, Germany), was supplemented with 3% fetal calf serum (Biochrom AG, Berlin, Germany), 50 IU penicillin/ml and 50 μg streptomycin/ml (Biochrom AG, Berlin, Germany). Tissue was kept under standard cell culture conditions in an incubator at 37 °C and an enriched atmosphere of 5% carbon dioxide. The culture medium was replaced every second day. A microscopic photograph was taken daily using a stereomicroscope (Stemi 508, Carl-Zeiss, Jena, Germany). The optic area of the IOL free of PCO cells was measured each day, until total confluence of the cells on the posterior capsule occurred, using time lapse microscopy ImageJ 1.8.0 (NIH, Bethesda, MD, USA).

### Immunofluorescence staining

After 30 days of cultivation in tissue culture, the capsular bag was washed three times with phosphate-buffered saline (PBS Dulbecco, Biochrom, Berlin, Germany) before being fixed in 4% paraformaldehyde overnight. After three washes with 0,1 M phosphate buffer (1:1 Na_2_HPO_4_ x 2H_2_O + NaH_2_PO_4_ x H_2_O) in distilled water, the IOL was extracted, and the zonules were carefully removed. The posterior capsule was divided into quarters, again fixed with entomological pins to a cell culture dish and incubated with blocking-Solution (3% bovine serum albumin, 0,1% triton X 100 in 0,1 M phosphate buffer) for blocking of unspecific antigen staining. Actin was stained without antibody by alexa fluor 488 conjugated phalloidin (Invitrogen, Carlsbad, CA, USA) for 120 min in a dilution of 1:100. A Cy3 conjugated antibody was used for 훼-smooth muscle actin (α-SMA) staining (1:50; **C6198,** Sigma-Aldrich, St. Louis, MO, USA) over night at 4 °C. Primary rabbit antibodies were used for fibronectin (F3648, Sigma-Aldrich, St. Louis, MO, USA) and collagen type 1 (ab34710, Abcam, Cambridge, UK) at a dilution of 1:50 over night at 4 °C. Following another three washings, slices were incubated with alexa 555 labeled goat-anti rabbit antibodies (1:500; A27039, Invitrogen, Carlsbad, CA, USA) for 120 min at room temperature. After three washings with 0,1 M phosphate buffer, the nuclei were stained using hoechst 33342 (Invitrogen) in a dilution of 1:2000 in 0,1 M phosphate buffer followed by additional three washings. The capsular bag was then whole mounted onto glass slides using antifade mounting for fluorescence medium (Vector Laboratories, Burlingame, CA, USA). Immunofluorescence staining was analyzed on an Axio Observer 3 (Zeiss, Jena, Germany) and documented by using the ZEN software (Zeiss, Jena, Germany). The expression of markers was graded subjectively by two independent researchers and graded from 0 (absent) to 3 (highly expressed).

### Statistical analysis

This was an exploratory study with limited resources (donor eyes). Therefore, a sample size justification was not deemed appropriate. Statistical comparison of PCO formation between different experimental groups was done using an ANOVA with Bonferroni post-hoc test and SPSS 25 (IBM, Armonk, New York, USA). For all analyses, *p* < 0.05 was considered statistically significant. All calculations were done in Excel (Microsoft, Redmond, WA, USA). Errors were presented as ±1 standard deviation. All graphs were created using Graphpad Prism 7 (GraphPad Software, La Jolla, CA, USA).

## Results

### Human capsular bag model

**Fig. 1:**
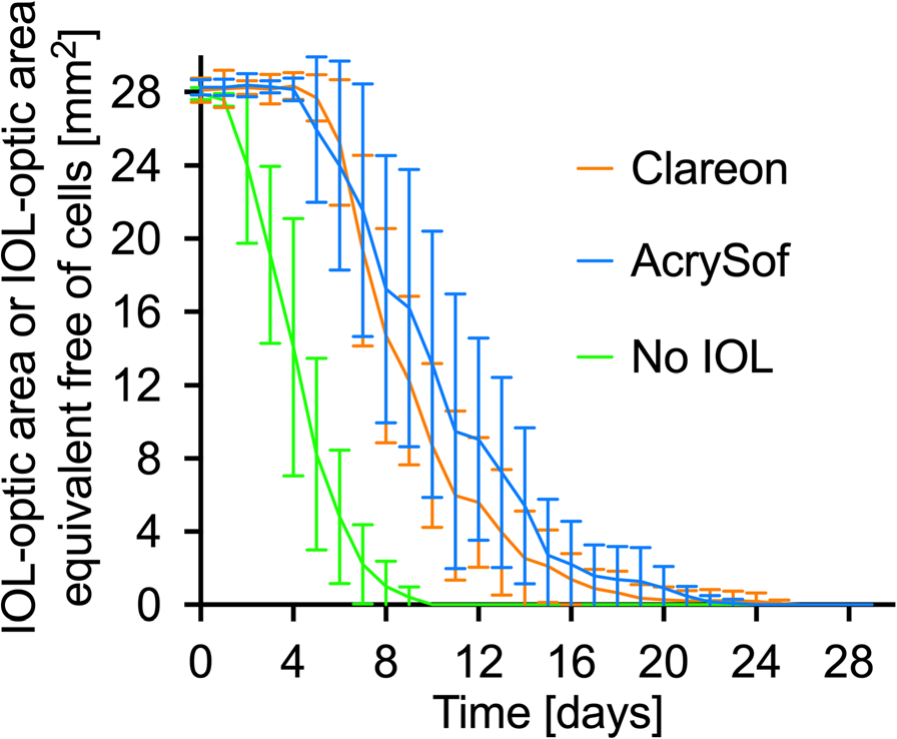
Measured is the area, which is free from PCO-cells on the posterior capsule below the IOL optic. In the control specimens without IOL implantation, an IOL optic area equivalent was used. This area was measured daily. Each colored line represents the mean of one experimental group

**Fig. 2:**
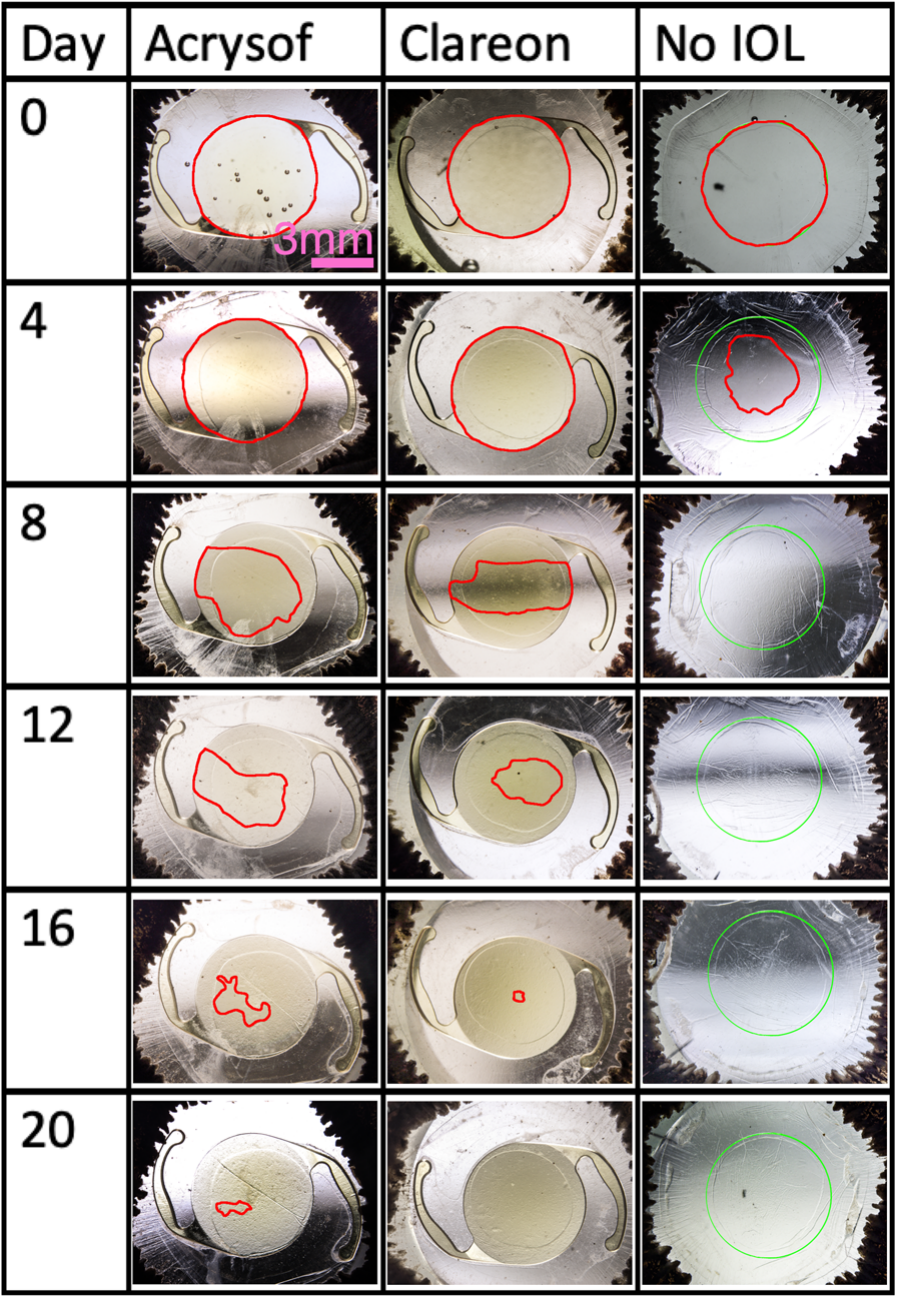
The growth pattern of the lens epithelial cells is summarized for the first 20 days (24 and 28 days are not presented as they did not add any extra information). The area below the IOL or the area equivalent (green circle) which is not covered by cells, is marked in red. The cells migrated from the capsule equator across the sharp edge into the center of the IOL. After full cell coverage was reached, fibrotic reactions caused contraction of the extracellular matrix with capsular folds to be formed. This effect was markedly increased in the capsular bag without IOL implantation

Both eyes of a donor were used, one was implanted with the AcrySof, the other with the Clareon IOL. Regarding the growth pattern, remaining lens epithelial cells started to grow from the capsule equator to the center of the IOL which, in vivo, represents the optical axis. After 6.5 ± 1.4 days, the cells migrated across the sharp edge in both IOLs. In the capsular bags without IOL, cells could be observed in the central optic area after 2.2 ± 0.8 days. After further cell migration and proliferation, full cell coverage of the posterior capsule in the experimental group without IOL implantation was observed after a mean of 8.6 ± 1.3 days. The implantation of an IOL could significantly increase this time by about two-fold to 15.7 ± 5.0 days (*p* = 0.005) in the AcrySof group and 14.8 ± 4.7 days () (*p* = 0.01) in the Clareon group. The difference between the two IOLs was not statistically significant (*p* > 0.99), which shows similar performance of the newer Clareon to the AcrySof IOL regarding the in-vitro formation of posterior capsule opacification (Fig. 1). After full cell coverage, the posterior capsule and the extracellular tissue was contracted producing capsular folds. This effect was observed to be markedly stronger in the capsular bags without IOL implantation and to be comparable between the AcrySof and the Clareon (Fig. 2).

### Immunofluorescence staining

**Fig. 3:**
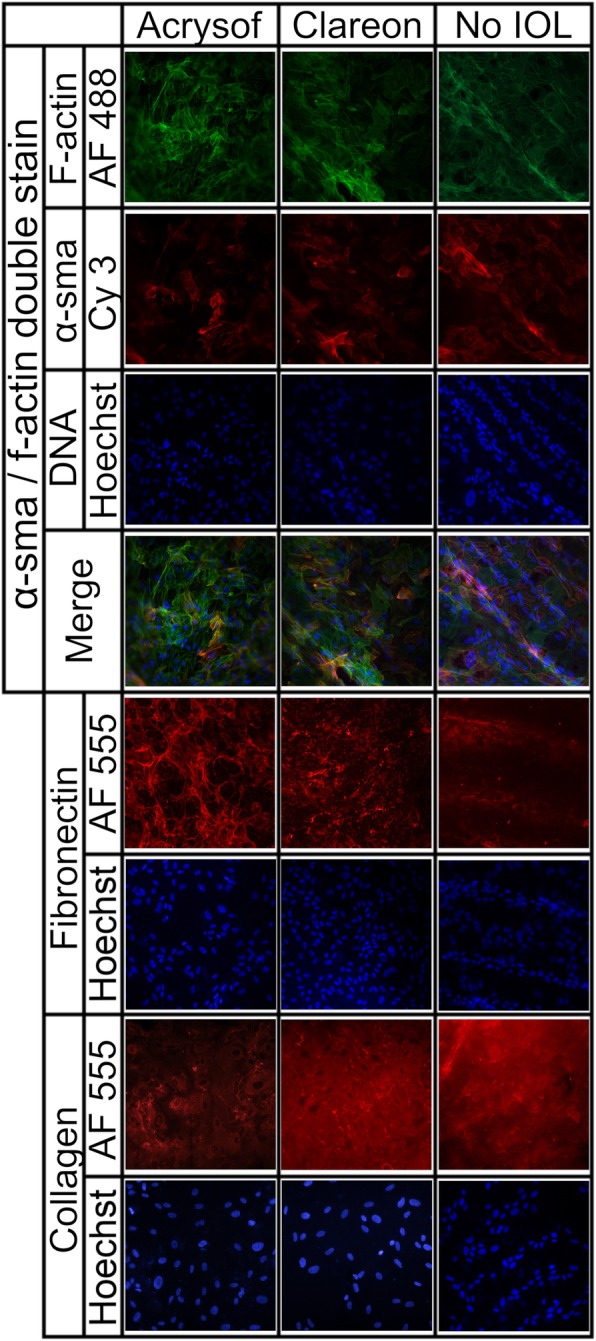
Representative immunofluorescence staining for f-actin, α-SMA, fibronectin and collagen type 1 of capsular bags without IOL and following AcrySof or Clareon IOL implantation. The double staining for α-SMA and f-Actin showed a partial co-localization of both markers and correlates well with a myofibroblast-like phenotype of the cells at the posterior lens capsule. For fibronectin focal and ribbon-like signals adjacent to cells at the posterior lens capsule were detected and were even more pronounced in the IOL groups. In the no-IOL group, fibronectin was predominantly located in folds of the posterior lens capsule and was hardly detected between the folds. For collagen type 1 a diffuse background staining was seen as it was secreted to the posterior capsule. In addition, in areas of the posterior lens capsule with a higher cell density or in capsule folds, an enhanced expression of collagen type 1 was observed. (magnification: 20-fold; blue, Hoechst staining)

Only cells on the central posterior capsule, which previously were located below the IOL, were evaluated, as this is the relevant pathological area for PCO. Cells on the posterior capsule of capsular bags with an implanted IOL showed a specific immunostaining for f-actin, α-SMA, fibronectin and collagen type 1, which showed no obvious differences between both IOL designs (Fig. 3). In contrast, the capsules without IOL implantation had mostly a different localization of markers and a different immunoreactivity when compared to that of an IOL implanted capsular bags.

In detail, for α-SMA, a specific filamentous intracytoplasmic staining was detected which was only partially co-localized with that of f-actin expressing cells in IOL implanted capsular bags. In contrast, in control capsules without IOL implantation, almost all cells on the posterior capsule assembling f-actin filaments showed α-SMA expression, suggesting a more pronounced myofibroblast-like phenotype of the cells. Further on, the signal for f-actin and α-SMA was more intense in cells from capsular bags without IOL treatment when compared to that of AcrySof or Clareon implanted capsules.

Following IOL implantation, fibronectin fluorescence appeared in higher amounts as focal points and around the plasma membrane. The capsular bags without IOL showed a more diffuse staining of the extracellular matrix for fibronectin, with the most prominent being in capsular folds. However, beside the folds, most cells had no immunoreactivity for fibronectin. This observation is consistent with the contractile activity of the cells, which is increased in the folds of the posterior capsule. In capsular bags which were cultured in the presence of an IOL, an increased signal for fibronectin around the lens epithelial cells was observed.

Collagen type 1 showed high background staining in all groups as it was secreted to the posterior capsule by the cells and was most prominent in folds of capsular bags without IOL implantation.

Overall, the enhanced expression of all markers except fibronectin in capsular bags without implanted IOL correlates well with the fibrotic response of cells in the capsular bags. However, in capsular bags, which had an IOL implantation, no obvious difference in the expression of fibrotic markers between the IOL types could be determined.

## Discussion

The new Clareon-IOL was developed with a precision sharp edge and a purported improvement in IOL material. Both, IOL material [18] and the square edge design [10], play a critical role in PCO formation. In our ex vivo study, we could not determine any differences in PCO formation of the newer Clareon when compared to the AcrySof IOL. Furthermore, there was a comparable fibrotic response regarding different molecular markers of fibrosis, which is in line with other reports [5]. In a recent meta-analysis of the AcrySof single and multi-piece models, data of three Alcon internal clinical trials on PCO formation of the Clareon were included [5]. Both the Clareon single-piece and multi-piece IOLs were investigated. Even though exact data of the trials are not presented, there were comparable results between the Clareon and the AcrySof Nd:YAG probability at 1 and 3 years, whereas only one of the three studies had three-year results. The authors concluded that the Clareon is likely to perform as well as, and possibly better than, AcrySof in terms of Nd:YAG capsulotomy rates. Furthermore, an in vivo rabbit study in fifteen New Zealand white rabbits with a follow up of 1 month did not detect a difference in PCO and anterior capsule opacification, as observed during clinical and pathological evaluation between the Clareon and the AcrySof IOL [19]. Our human ex vivo data in the capsular bag model shows a comparable result.

In comparison to other currently available IOL models, the AcrySof model has shown a low incidence of PCO in many thousand eyes in several population based studies and meta-analyses [5, 20]. Our study and other aforementioned, previous studies have shown a comparable performance of the Clareon and the AcrySof. This might be due to the precision edge of Clareon, which features a square posterior optic edge similar to that of the AcrySof. Even though the results show comparable PCO rates, the manufacturer claims potential advantages of the Clareon regarding less glistening, less axial displacement and reduced dysphotopsias and glare.

Myofibroblastic cells are a hallmark of PCO formation. Epithelial mesenchymal transformation of lens epithelial cells [21] or a direct, myogenic cell precursor called the Myo/Nog cells, are thought to be the origin of those myofibroblasts [22]. Several growths factors and cytokines can cause their activation, leading to cell growth, migration, fibrosis and contraction of the posterior capsule [3]. In order to be able to cause this unwanted wound healing reaction, cells have to express certain proteins and cellular behavior, which are not present in healthy lens epithelial cells. α-SMA is one of the proteins involved in contraction of cells and is usually present in human explanted post-mortem capsular bags with PCO [23] and implicated in PCO formation [24]. In our experiments, we detected an equal amount of α-SMA positive cells in the groups with IOL implantation and cell presence was enhanced in empty capsular bags without IOL. Hence, the implantation of an IOL seems to block the formation of epithelial-mesenchymal transition of cells at the posterior lens capsule. Collagen type 1 is a secreted protein and produced by lens epithelial cells when undergoing fibrotic reactions [25] and is therefore also a marker for myofibroblastic activity. Collagen type 1 was visible extracellular and on top of the posterior capsule and especially in the capsular folds seen in the group without IOL. To our knowledge collagen type 1 is not found in the healthy capsule [26], yet previous post-mortem human studies have shown that it is highly abundant in the fibrous extracellular matrix typically found in PCO (e.g. Elschnig pearls and Soemmering ring) [27]. Furthermore, it can accelerate fibrosis by direct interaction with lens epithelial cells through metallo-matrix proteinases in lens epithelial cells [28]. This might explain why its presence is higher in the capsular folds, where fibrosis is increased. Myofibroblasts furthermore overexpress fibronectin, which allows them to bind to extracellular matrix. Fibronectin modulates and stabilizes the extracellular matrix in wound healing processes [29]. Fibronectin was present in the expected location extracellularly and enhanced below the IOLs and in the capsular folds, where increased fibrotic cell activity is suspected [21].

Limitations of this study include the experimental laboratory character and the use of fetal calf serum (FCS). The human capsular bag cells do still actively synthesize proteins and growth factors which accelerates their growth following more than 1 year in protein-free medium in cell culture [30]. The finding that persistent cell proliferation can occur is significant, as it suggests that lens epithelial cells have a baseline ability to remain active and contribute to PCO development, which typically becomes clinically relevant years after surgery [31]. Due to a many month-long incubation of the capsular bags being out of scope for this study, we added FCS, which contains various growth factors, to accelerate epithelial mesenchymal transition and proliferation of the cells. The scientific community is currently still in discourse on what is the appropriate amount of FCS for the capsular bag growth model. The used percentage varies widely from 0 to 10% across the literature and depends on the scientific requirements and the hypothesis [12, 30]. The higher the concentration of FCS, the faster the PCO formation. In contrast, individual factors like donor age contributing to PCO formation are less relevant to the present analysis, since only donor pairs of eyes were used to compare PCO formation between both IOLs. After careful consideration we choose 3% FCS as it is still high enough to allow individual donor factors which seems more clinically relevant.

## Conclusion

Our study clearly demonstrates that the new Clareon IOL shows no difference in terms of PCO formation when compared to the AcrySof IOL in the human ex vivo capsular bag model. Cellular growth was comparable, and all tested fibrotic markers did not vary between the two IOLs. Both IOLs had a protective effect on PCO development after implantation when compared to capsular bags without IOL implantation.

## Data Availability

The datasets used and/or analyzed during the current study are available from the corresponding author on reasonable request.

## Abbreviations

FCS: Fetal calf serum
IOL: Intraocular lens
PCO: Posterior capsule opacification
α-SMA: Α-Smooth Muscle Actin
